# Design and Implementation of Fast Spoken Foul Language Recognition with Different End-to-End Deep Neural Network Architectures

**DOI:** 10.3390/s21030710

**Published:** 2021-01-21

**Authors:** Abdulaziz Saleh Ba Wazir, Hezerul Abdul Karim, Mohd Haris Lye Abdullah, Nouar AlDahoul, Sarina Mansor, Mohammad Faizal Ahmad Fauzi, John See, Ahmad Syazwan Naim

**Affiliations:** 1 Faculty of Engineering, Multimedia University, Cyberjaya 63100, Malaysia; hezerul@mmu.edu.my (H.A.K.); haris.lye@mmu.edu.my (M.H.L.A.); nouar.aldahoul@live.iium.edu.my (N.A.); sarina.mansor@mmu.edu.my (S.M.); faizal1@mmu.edu.my (M.F.A.F.); 2 Faculty of Computing and Informatics, Multimedia University, Cyberjaya 63100, Malaysia; johnsee@mmu.edu.my; 3 IPTV Development, Unifi Content, Telekom Malaysia Berhad, Cyberjaya 63100, Malaysia; asyazwan@tm.com.my

**Keywords:** foul language, speech recognition, censorship, deep learning, convolutional neural networks, recurrent neural networks, long short-term memory

## Abstract

Given the excessive foul language identified in audio and video files and the detrimental consequences to an individual’s character and behaviour, content censorship is crucial to filter profanities from young viewers with higher exposure to uncensored content. Although manual detection and censorship were implemented, the methods proved tedious. Inevitably, misidentifications involving foul language owing to human weariness and the low performance in human visual systems concerning long screening time occurred. As such, this paper proposed an intelligent system for foul language censorship through a mechanized and strong detection method using advanced deep Convolutional Neural Networks (CNNs) and Recurrent Neural Networks (RNNs) through Long Short-Term Memory (LSTM) cells. Data on foul language were collected, annotated, augmented, and analysed for the development and evaluation of both CNN and RNN configurations. Hence, the results indicated the feasibility of the suggested systems by reporting a high volume of curse word identifications with only 2.53% to 5.92% of False Negative Rate (FNR). The proposed system outperformed state-of-the-art pre-trained neural networks on the novel foul language dataset and proved to reduce the computational cost with minimal trainable parameters.

## 1. Introduction

Filtering audio and video content has become a social concern with the high exposure of many young adults to portable and immediate screen time sources. There is a risk a person could be exposed to large amounts of offensive and foul language incorporated within entertainment videos and movies displayed at the online platforms and broadcasting channels. For example, almost all movies contain foul word and have kept increasing through the years [1], while foul language is known to bring a negative effect to the society [2]. Furthermore, broadcasting companies and media-sharing platforms have been held accountable to provide appropriate content through censorship tools. Censorship is a complex phenomenon in filtering and providing language content worthy of viewers due to the constraints in personnel, cost, time, and human fatigue that could lead to misdetection of unwanted content. The proposed study aimed to construct an astute, competent, and mechanized censorship system in identifying undesirable spoken terms (profane language) within audio signals, such as stand-alone audio files and signals assimilated in prominent and accessible video-sharing websites and broadcasting media. In this regard, neural networks facilitated audio censorship in videos (movies and entertainment shows) and reported intriguing characteristics of the techniques.

Speech recognition through deep learning has recently gained popularity. Specifically, speech identification systems operated by identifying various utterance types (spontaneous and continuous speeches and connected and isolated words) [3]. This study served to build an astute censorship system to precisely identify unfavourable speech content, given that literature on intelligent speech identification models using deep learning has solely emphasized inoffensive language identification [4]. For example, recent studies have used conversational and read speech dataset that are clean of foul language utterances such as LibriSpeech [5] Wall Street Journal (WSJ) corpus [5,6], Google’s voice search traffic dataset [7], Google commands dataset [8,9], and speech emotions dataset of conversational speech dialogues [10]. A recent study has emphasized the identification of foul language using pre-trained CNNs (e.g., Alexnet and Resnet50) [11]. However, proposed solutions suffer two issues of detection rate and complex computational cost with a large number of network parameters, which does not suit applications of real time monitoring for foul language filtering in videos. Hence, this research proposed profane language recognition using two novel, lightweight, and distinct neural networks.

Generally, speech and acoustic-based recognition methods use a combination of feature extraction of acoustic features techniques and various classifiers. Mel-Frequency Cepstral Coefficients (MFCCs) are common and mostly use feature extraction technique for speech and acoustic signals and have been in various applications such as audio-visual classification of human actions utilizing the frequency features of action’s sounds [12] and urban sound classification to detect the human’s environments sounds [13]. Spectral features mapping approaches have been also used for large scale Automatic Speech Recognitions (ASR) [14]. Additionally, Linear Prediction Coefficients (LPC) and Linear Spectral frequencies (LSF) were used for different applications like speaker recognition [15], spoken digits recognition [16], and emotion recognition from speech [17]. Discrete Wavelet Transform is another feature extraction technique that has been used for speaker recognition [18], speech semantic and emotions recognitions [19,20]. Furthermore, spectrogram images are the best choice of speech and acoustic feature extraction that is suitable for CNN models. Spectrogram feature extraction approach has been used for foul language classification with pre-trained CNN networks [11]. Various classification and detection tasks utilize spectrogram features like Speech emotion recognition [21,22], acoustic scenes and sound events recognition [23,24].

The main classifiers used for acoustic and speech recognition include Hidden Markov Model (HMM), Support Vector Machine (SVM), CNNs, and RNNs. Support Vector Machine classifier has been used for several classification tasks related to acoustic features like speech emotions recognition [25] where speech features were extracted using MFCCs and SVM implement the task of emotions classification based on speech features extracted. CNNs are powerful networks on image prediction with the power of convolution and filtering of network layers. CNNs have been used for the detection of acoustic features from images of speech spectral features like speech emotions recognition [21] and sound events classification [23] where CNNs demonstrate a high efficiency of image predictions. CNNs have also been used for large scale speech recognition models as a feature extraction layer [26]. RNNs are power neural networks for serial inputs with memory cells like LSTMs. RNNs have been used for different applications like spoken digits recognition [27], acoustic scenes classification and filtering [28], and screams detection [29]. A combination of HMM and RNN hybrid models are used for large scale automatic speech recognition [5,6].

The first study challenge involved the data scarcity of profane language. Hence, a new database on profane utterances from various accents was constructed for an authentic identification system. Additionally, all the recordings and data samples derived from the videos were genuine without simulations through acoustic mixes or synthetic signals. The background noise used during the data augmentation process could be a sound source apart from speech. The additional derivations were supplemented to ensure the sturdiness of the system involving noise-filled environments and audio signals with multi-sources. The second aspect concerned uneven data samples as verbal offensive language data samples were notably lesser than normal utterances.

This study applied acoustic MFCCs and Mel-spectrograms on two deep-learning structures, namely, CNNs, and RNNs using LSTM cells to identify pre-segmented foul language samples. A reciprocal categorization task was conducted comprising nine profane words and normal conversational speech. Another set of 10 classes (with further divisions of the foul class into nine sub-classes) was analysed through 10-way classifications. The study also discussed noisy data and data augmentation implications. The remaining study sections are arranged as follows: Section 2 describes the study database, Section 3 presents the methodological background of neural networks and temporal feature extractions, Section 4 highlights the study parameters and contexts, Section 5 presents the study findings, and Section 6 reveals the study conclusion and implications.

## 2. Database 

This study presented a unique dataset (the MMUTM foul language dataset) obtained and analysed at Multimedia University, Malaysia for a film censorship research project in collaboration with Telekom Malaysia (TM) [11]. The dataset was a selection of profane language collected through the recordings of various areas and environments to verify the strength of the recommended model during system assessment. The dataset was recorded by asking 117 volunteers to utter nine different offensive words. In some instances, only one utterance was recorded, while the highest number of utterances recorded by a speaker was 10 for each of the nine foul words. Besides, the dataset comprised manually-retrieved and natural data samples from random videos to increase the sample variations that contributed to the dataset complexities.

The nine categories of profanity include “Asshole”, “Balls”, “Bastard”, “Bitch”, “Cock”, “Cunt”, “Dick”, “Fuck”, and “Pussy” as offensive language or profane content to be filtered under the Malaysian Film Censorship Act 2002 [30]. Regardless, the derivation of the aforementioned classes posed study complications regarding offensive language identification. The following abbreviations substitute the profanities for acceptable sample depictions throughout this paper: “A: asshole”, “B: balls”, “Ba: bastard”, “Bi: bitch”, “C: cock”, “Cu: cunt”, “D: dick”, “F: fuck”, and “P: pussy”. A normal class was incorporated into this experiment in representing casual speech and distinguishing profane words from normal counterparts during censorship. The normal samples were obtained from Freesound, a sharing site for audio samples [31]. Additionally, the normal class is categorized as “N” in the following sections. The database properties were set at 16-bits PCM, whereas the 1-channel samples were set at 16-kHz. 

### 2.1. Data Labelling

The study data were manually labelled in two stages. The first stage involved labelling the complete dataset into two-category classifications (foul versus normal), whereas the second stage involved decomposing the foul class into nine sub-classes for a particular annotation. The definition of offensive data sub-classes potentially enabled precise profanity identification as opposed to a two-category classification (foul versus normal).

The dataset consisted of three sets: training, validation, and test sets for five-fold cross-validation under the speaker-independent approach, wherein the assessed and verified sample utterances were derived from speakers who differed from the training samples. Train sets were used to train the model on the target application of foul language recognition, validation samples were used to validate and test the model alongside algorithm training phase to ensure accuracy performance convergence and avoid underfitting and overfitting, while a test set was used to evaluate the trained model performance after training completion and report the performance measure results. Table 1 presents the distribution of 3105 offensive language samples and 5100 normal samples across training, validation, and test sets for each main class and sub-class. Under the foul category, 3105 samples comprised of 345 data points for each of the nine subclasses. Despite the availability of a large normal sample, only a portion of the normal dataset was selected to alleviate the data sample variations due to data disparity between normal samples as a class and each profanity sub-class. For example, the training set for each foul class consisted of 207 samples, while the normal train set consisted of 3060 samples. This study resembled authentic scenarios or video and audio files, whereby the number of foul words was notably lesser than the normal words within a casual speech.

### 2.2. Data Augmentation

Data augmentation was utilized in this study to increase the dataset quantity for systems resembling speech identification and image processing [32]. Large datasets were employed to increase the precision of voice recognition systems, particularly for deep neural networks. Regardless, the absence of a large dataset could be rectified by increasing the dataset size. As the human voice differed in terms of pitch and volume, both aspects could be refined for a larger dataset. Given that pitch corresponded to frequency, frequency alterations would transform the pitch dataset through dataset amplification. Additionally, voice data could be augmented using various techniques, such as tempo and speed perturbation [33].

Insufficient data for censorship implementation hindered the construction of foul language identification models. Hence, the MMU foul language database was expanded to increase the dataset and elevate the model quality and robustness. Specifically, the study dataset was augmented nine times (see Table 2) by incorporating noise (three natural background noise types and white noise) and manipulating sample’s frequency and pitch.

## 3. Methods

This study employed deep learning networks, particularly CNNs and RNNs through LSTM cells. Furthermore, MFCCs were utilized as a temporal structure extraction technique apart from Mel-spectrogram images in sequencing feature representations of audio samples. Thus, this section elaborates on the fundamentals of CNNs, RNNs, MFCCs, and Mel-spectrograms in the study.

### 3.1. Artificial Neural Networks (ANNs)

The ANNs were extensively researched and employed as an influential method for regression and classification problems involving multiple applications. Following the specific descriptions in relevant works of literature [34,35,36,37,38], ANN output in a classification paradigm was an estimate y(n) of the posterior probability p (c | x(n)) of each class c ∈ [1, C]. Specifically, C denoted the number of classes and was given an input data of vector x(n), wherein n defined the time index. As such, the selected class portrayed the highest posterior probability. Additionally, ANNs were linked to supervised learning through labelled data to appropriately adjust the weights for authentic information.

#### 3.1.1. Recurrent Neural Networks

The RNN is a type of ANN with high potential in analysing temporal sequence inputs through memory cells underlying the RNN structure. The RNN forms include (i) unidirectional, wherein the knowledge permeating memory cells advances in time, and (ii) bidirectional, wherein information is reciprocal (moves forward and backward in time) [39]. On another note, LSTM involves unique cell types that elevate the required problem-solving skills for long-term temporal dependencies. Therefore, the vanishing gradient problem following the exponential decline or loss of gradient function was attested. In the study context, LSTM pertained to distinct cell types with special units supplemented to the standard units. Moreover, memory was built using the forget gate for long-term memory retention [40].

The LSTM predicted the current time following all the past time inputs. For each layer, LSTM computes at time (*t*) as follows:

(1) $$f_{t}=\sigma_{g\;}\left(W_{f}x_{t}+U_{f}h_{t-1}+b_{f}\right)$$

(2) $$i_{t}=\sigma_{g\;}\left(W_{i}x_{t}+U_{i}h_{t-1}+b_{i}\right)$$

(3) $$o_{t}=\sigma_{g\;}\left(W_{o}x_{t}+U_{o}h_{t-1}+b_{o}\right)$$

(4) $$c_{t}=f_{t\;}c_{t-1\;}+i_{t\;}\sigma_{c\;}\left(W_{c}x_{t}+U_{c}h_{t-1}+b_{c}\right)$$

(5) $$h_{t}=o_{t\;}\sigma_{h\;}\left(c_{t}\right)$$

Specifically, $\sigma_{g\;}$ = sigmoid function; $\sigma_{c\;}$and $\sigma_{h\;}$= hyperbolic tangent functions; *W* and *U* = weights; *b* = bias; *x* = input vector; while *f*, *i*, and *o* = gates; *c* = internal cell states; and *h* = hidden or recurrent states. In this vein, LSTM only employed past information, wherein a forward pass obtained feature-maps following Equations (1)–(5). 

#### 3.1.2. Convolutional Neural Networks

The CNN model consisted of stacked convolutional and fully-connected layers with sub-sampling or pooling layers in between. Furthermore, CNNs are easily trained with lesser parameters compared to fully-connected networks with an equal number of hidden units. Additionally, CNNs functioned through multiple filters in horizontal and vertical lines over an image for various signal identifications. The signals consequently enabled the mapping of different image feature portions and trained classifiers on the target application. The convolution layers extracted features from an input image and sustained the pixel relationships by acquiring image features through small squares of input data. Furthermore, the extraction employed a mathematical operation involving two inputs (image matrix and a filter or kernel). Image convolutions using various filters could also perform specific operations, such as fault diagnosis [41], image encryption [42], edge detection, blurring, and sharpening through filter applications [43].

Pooling layers would reduce the parameters of a given image. For example, although spatial pooling (also known as sub-sampling or downsampling) reduced the dimensionality of each map, vital information was retained. Furthermore, spatial pooling could be categorized into (i) max pooling, (ii) average pooling, and (iii) sum pooling. For example, max pooling selects the largest element from the corrected feature map, while the sum of all the feature map elements are recognized as sum pooling. The fully-connected layer comprised a flattened matrix vector under the convolution and pooling processes. The layer resembled a neural network that integrated the convolution process features to construct a model. An activation method involving SoftMax or sigmoid can be applied to classify outputs following the desired application. SoftMax function converts a vector of N values into a vector of N values that sum to 1. SoftMax transform any input with zero, positive and negative values into values between 0 and 1, so that they can be interpreted as prediction probabilities [43].

### 3.2. Temporal Sequence Features

The target speech is generally characterized by spectral content represented through the MFCC vectors and Mel-spectrograms. The vectors were applied by sliding an analysis window over a portion of the size to ensure overlapping between frames. Regardless, the coefficient vectors representing a given speech differed unpredictably following the duration and characteristics of the target speech [44]. The CNNs structures assessed visual inputs through temporal dimension, whereby 2D Mel-spectrograms were consecutively established through coefficient vectors for CNNs. Consequently, the convolution process extracted the complete spectral content features from frequency and time domains, while RNNs complemented speech sequences following the capacity to analyze input vector sequences through memory by permitting the network to identify the consecutive audio patterns. The memory cell enables the network to analyze successive input vectors over a long period without an arbitrary segmentation despite the vector-by-vector input into RNNs.

The temporal sequence features are represented by a vector of features extracted from speech and acoustic signals. Serial vectors are extracted with MFCCs and Mel-spectrograms. Feature extraction technique basically includes a few steps to create the signals representations. Pre-emphasis is the first step for feature extraction approach, which refers to filtering that emphasizes the higher frequencies to balance the spectrum of voiced sounds that have a steep roll-off in the high-frequency region. Windowing then is used to divide the input signal into small frames with overlap window to ensure all serial sample features are extracted. Windowed parts then are used to apply Discrete Fourier Transform (DFT). Then, taking the log of the magnitude, warping the frequencies on a Mel scale, and then applying the inverse Discrete Cosine Transform (DCT) to produce the Mel-frequency sequence features. 

## 4. Experimental Setup

This section elaborates on the experimental procedure following the data collection and annotations previously mentioned in Section 2. 

**Figure 1:**
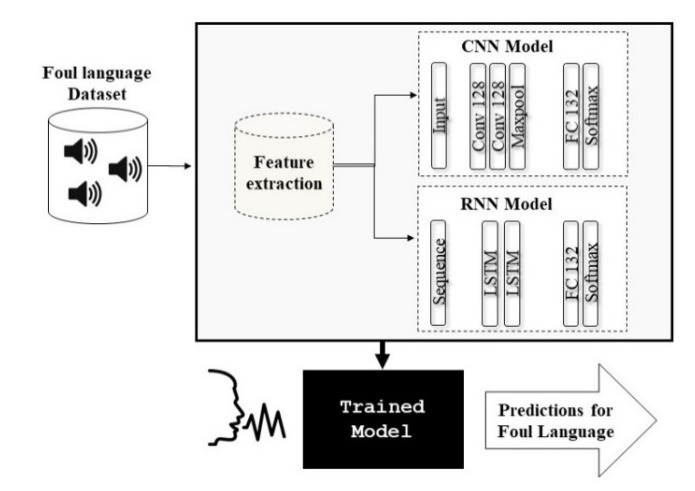
Process flow of foul language detection model.

Figure 1 presents the recommended system for profane language detection. The study dataset was analysed for feature extraction with Mel-spectrograms and MFCCs. Extracted features were then used to train two different models separately. Hence, the two models were not fused for decision fusion in this study. RNNs with LSTM cells were trained with MFCCs features, while CNNs were trained with Mel-spectrogram images. Each of the trained models were then assessed separately with the test features of speaker-independent isolated utterances for potentially offensive language classification outputs. This study recommended two models for offensive language identification (RNN and CNN) and compared the performance of the two proposed models. Each model was trained and assessed for two problems: two-class and 10-class identifications of profane language. Besides, the experiment was performed using k = 5 cross-validation for a strong model evaluation. The models were also trained and assessed with authentic and augmented data to study the effect of the utilized data and noise on RNN and CNN performance. The experiment was repeated to assess the suggested model using various test dataset samples: clean and noise-filled samples with signal-to-noise ratios (SNRs) of 20 dB, 10 dB, and 0 dB. 

### 4.1. Feature Extraction

**Figure 2:**
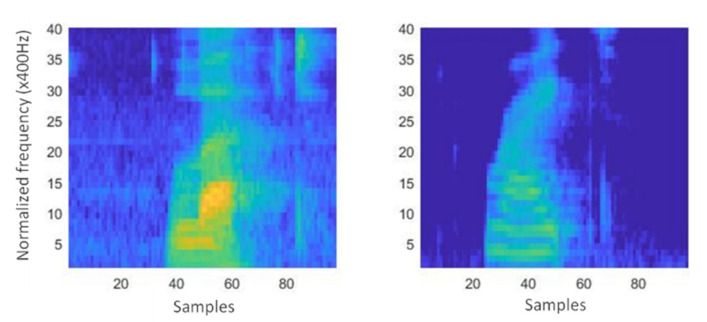
Spectrogram samples of foul language (40-by-97).

The MMUTM foul language dataset utilized 16-bits PCM and 1-channel samples at 16-kHz that was subsequently converted into a series of feature vectors. The RNN acoustic feature vectors comprised 42 MFCCs that were computed every 10 ms with a window length of 20 ms, hence resulting in a 10 ms overlap window. Given the aforementioned parameters, 100 feature vectors were generated every second using 42 MFCCs. In contrast, 42 log Mel-frequency spectrogram coefficients were utilized to define the visual representations of speech energies as frequency spectrums. Offensive and normal speech spectrograms were analysed using the following parameters: 1-second segment duration, 0.025 frame duration, 0.010 overlap window between frames, and 40 frequency bands. Additionally, the produced Mel-spectrogram images were 40-by-97 in size. Figure 2 presents foul language spectrogram samples between two foul utterances.

### 4.2. Network Architectures

Based on Figure 1, two strategies were applied to distinguish offensive language from the casual counterpart. Initially, the foul language features were not specifically considered: The profane words were labelled under the foul class (foul versus normal). The foul class was then divided into nine sub-sets under the same category (nine profanities versus normal class). Regarding RNN, a configuration of two hidden layers was employed with 512 and 256 units per layer for the first and second hidden layers, successively. Table 3 presents the CNN architecture for offensive language identification. 

The suggested CNN model comprised five convolutional layers, five ReLU layers, and four max pooling layers. The last layers (fully-connected and SoftMax) were employed to incorporate Mel-spectrogram images into the target application. Table 3 also outlines the fully-connected layer with outputs of 2 (10), thus indicating that both the problems experimented on were two-class and 10-class.

### 4.3. Training Algorithm Settings

All the models were trained using the momentum technique (Adaptive Moment Estimation). In contrast, cross-entropy was employed as the loss function. In total, 64 input matrices and a learning rate of 0.001 for 70 epochs were utilized for CNNs. Contrarily, RNNs employed Unidirectional LSTM units that were trained on 50 to 80 epochs with a learning rate of 0.01 and 64 sequential samples. Notably, this study did not employ pre-training as all the profane language database networks were trained from scratch. The models were trained and assessed using five varying folds for the authentic averaging outcomes of complete data samples. Initially, the models were trained on a two-class problem (foul versus normal) and 10-class problem to explicitly define and detect the foul language type. We use TensorFlow framework to develop, train, and test our models. Experiment implementation was carried out using Alienware desktop computer with Windows 10 (64-bit), 64 GB RAM, Intel Core i7-8700 CPU @ 3.20 GHz, and an NVIDIA GeForce GTX 1080 Ti. 

### 4.4. Metrics

Evaluation metrics were computed based on the confusion metrics deduced following each experiment. The suggested models were then verified and assessed with five varying folds for accuracy. The average outcomes concerning folds k = 1 to 5 are presented in the next section. Specifically, the offensive language model performance was assessed under Accuracy, Precision, F1-score, True Positive Rate (TPR), False Positive Rate (FPR), and FNR as follows:

(6) $$Accuracy=((N_{tp}+N_{tn})/N_{total\;})\times 100$$

(7) $$TPR=R=(N_{tp}/\left(N_{tp}+N_{fn}\right))\times 100$$

(8) $$FPR=(N_{fp}/\left(N_{fp}+N_{tn}\right))\times 100$$

(9) $$FNR=(N_{fn}/\left(N_{fn}+N_{tn}\right))\times 100$$

(10) $$F1-score=\left(\frac{2\left(P\times R\right)}{\left(P+R\right)}\right)\times 100$$

F1-score computed under precision (*P*) and recall (*R*):

(11) $$P=(N_{tp}/\left(N_{tp}+N_{fp}\right))\times 100$$

In the study context, *N_tp_*, *N_fp_*, *N_fn_*, and *N_total_* denoted the number of true positives, false positives, false negatives, and total samples in all the segments.

## 5. Experimental Results

Two ANNs were investigated in this study to distinguish the profane language for automated censorship purposes. The results revealed an average of five cross-validations to generate an authentic outcome throughout the dataset. Additionally, the results were derived from pre-segmented training and testing data samples. The experiments were also performed using speaker-independent mode. Several techniques were utilized to avoid model overfitting including cross validation where we divided the dataset into five folds. The models were trained on four folds and tested with one fold at each run. Then, models were evaluated using the average metrics of the five run metrics to produce a generalized evaluation and avoid overfitting. Data augmentation were used to increase the data size of the nine foul classes. Additionally, proposed models were designed to avoid model complexity as well as avoiding overfitting. Therefore, errors were minimal for both training and test phase.

### 5.1. Performance of Two-Class Models

Table 4 presents the two-class model performance using CNN with 5589 augmented offensive utterance samples and 9180 augmented normal speech samples. Table 4 also presents the standard deviation for performance measures. Following Table 4, CNN performed positively in the categorization of profane and normal language with low FNR and FPR. For example, the foul class achieved 2.19% and 3.88% for FPR and FNR, respectively. Regardless, the model performance was slightly improved for the normal speech class with 2.17% of FPR and 1.17% of FNR. Thus, a wider range of normal speech compared to the offensive language dataset (involving only nine varying sub-classes) was identified. The F1-score measured both P and R in TPR concerning the CNN model performance for an uneven dataset. Consequently, the CNN model achieved the best average performance in offensive language classification for the two-class problem with a high F1-score of 96.92% and 98.39% for the foul and normal classes, respectively. 

Table 5 demonstrates the two-class model performance using RNN with LSTM cells using performance measures and standard deviation. Based on Table 5, the RNN model performed positively in categorizing foul and normal language. The foul class achieved 4.88% and 0.83% for FPR and FNR, respectively. In contrast, the RNN model performance portrayed varying patterns on P and TPR. Specifically, the normal class had a higher P of almost 1.83% over the foul class, whereas the foul class had a higher TPR of 6.05% over the normal class. In this vein, the foul class reflected a minimal error in categorizing foul samples as a normal sample and a higher error in categorizing normal samples as a foul sample. Nevertheless, the F1-score measured both P and R in TPR concerning the RNN model performance. As such, the RNN model achieved an average performance (97.81% in F1-score) in offensive language identification. 

Overall, both models consisted of close performance metric values albeit a minute difference for two-class problem. Although sequential speech inputs highlighted better RNN performance in identifying temporal data with ongoing audio streams, CNN outperformed RNN in pre-segmented data sample identification following the feature extraction and visual classification in CNN. Hence, the best probability match under the detailed features extracted by the speech Mel-spectrogram image filters was detected in CNN.

### 5.2. Performance of 10-Class Models

The performance assessment of the proposed models on all 10 classes (nine profanity classes versus normal class) is presented in Table 6 and Table 7. The model test was performed with data disparity and revealed an intriguing pattern. Specifically, the nine-class profanity model was assessed using 621 utterances for each foul class. The experiment was conducted to accurately identify foul utterances instead of a “foul” class. The primary finding from Table 6 and Table 7 revealed that nine of the 10 sub-classes obtained an FNR below 10%, whereas one sub-class achieved an FNR of less than 12% for both models.

Following the 10-class problem outcomes, it was reported that the models generated good F1-scores for all 10 classes (exceeding 92% for CNN and 91% for RNN), thus implying positive sensitivity and specificity in offensive language detection. Contrarily, CNN reflected slightly higher performance metrics over RNN in class detection excluding class “P”, whereby both models demonstrated the same FPR of 0.40%. Class “F” obtained the lowest metric with a high FNR of 10.00% and 11.30% for CNN and RNN, respectively. In this regard, a large “F” class portion sample was misclassified with other classes regarding the division of a one-foul class into nine different classes, specifically the classes reflecting similar acoustic features (“C” and “D”). Overall, the average performance of the 10-class problem indicated that CNN outperformed RNN with a minute difference in metric values. 

In Section 5.1 and Section 5.2, the two-class system reflected better performance than the 10-class model comprising the same architecture and data folds. For example, RNN obtained an average of 3.86% in FNR for the two-class problem and an average of 5.19% in FNR for the 10-class problem. Additionally, the average F1-score in RNN decreased from 96.73% for the two-class problem to 94.85% for the 10-class problem. The decline could be analysed using the confusion matrix in Table 8. Notably, several profanities from the nine classes were misclassified as other offensive words, whereas the two-class problem fell under one “foul” category. Table 8 presents a confusion matrix of 10-class problems with RNN using one of the folds to outline the misclassification between profanities. Although 10-class models perform less better compared to two-class models, this study proposes the use of a different class model to precisely detect the exact spoken profane terms. The need for exact term recognition comes from the applications these models are to be used for, which are censorship and content moderation. Foul words have several levels of restrictions for videos and audio streams, which differ from country to another. For example, the same word class could be allowed for viewing at some regions, while it is restricted in some other regions. Additionally, some content ranking like shows for adults may allow the appearance for such profane words, while it is totally restricted for shows and videos attended by children. 

### 5.3. Discussion and Comparative Analysis

Speech detection models concern two issues that include performance and computational cost. Model’s performance and computational cost are trade-offs for different applications. A recent research has produced fine-tuned pre-trained CNN models for the foul language recognition on the MMUTM foul language dataset [11], but the cost complexity is very high. In this research, we addressed these issues and designed different novel models for the foul language recognition, however, CNN architectures outperformed RNN architecture. Hence, the CNN model was compared to the state-of-the-art models for profanity recognition. Our proposed CNN model utilized fewer layers that reduced the time complexity while achieving good performance metrics. Table 9 details the comparative analysis based on F1-score (comprising precision and recall), FNR, total parameters, and test time of our CNN model and baseline algorithms of a recent study on MMUTM foul language dataset that utilized finetuned Alexnet, VGG16, GoogLeNet, and Resnet50.

In Table 9, we showed the outperforming results of the proposed system based on F1-score, FNR and network parameters, which is significantly better than other systems, where the proposed model outperformed the best baseline algorithms with 1.57% FNR and F1-score of 1.91%. We developed a lightweight CNN model for foul language recognition with high performance and reduced cost complexity, which is suitable for monitoring the real-time applications and censorship. The current model has a total parameter of only 57 K, while GoogLeNet with the least number of parameters amongst baseline algorithms has almost 6 M parameters. The proposed system requires parameters of only 1/105 of the parameters required for GoogLeNet and lesser parameters requirements compared to the other baseline algorithms. Additionally, the proposed CNN model and baseline algorithms were tested for prediction speed with GPU and CPU separately to evaluate the model response on different hardware resources. Notably, the proposed model outperforms baseline algorithms by a huge margin in terms of prediction time per sample with only 2.05 ms and 2.33 ms using GPU and CPU, respectively. Thus, computational cost and prediction time minimization has been achieved. 

The proposed CNN model for the foul language recognition consists of modified filters in convolutions and pooling layer. Our model recognizes the spoken terms from the frequency pixels with reduced model size and simple architecture of a few layers with novel filter shapes. We used lower dimensions of spectrogram images to suit the simple CNN architecture and maximize the feature extraction efficiency. Therefore, dimensionality was reduced, cost complexity was reduced, and promising results were achieved.

This work was conducted on a novel dataset with an imbalance between foul classes and normal conversational speech, which mirrors the natural distribution of the classes in human speech and words utterances in videos and audio files; this could be a way for the classifier to deal with the natural imbalance between the classes considered. However, it is true that it could be an issue in learning a representation of each class; the limitation is that the model performance drops on classes with lesser samples as in the case of dividing foul class into nine classes. Division of foul class into nine classes corresponds to reducing the number of samples per class. In the context, foul class is nine times larger than each of the foul subclasses, which produce a high volume of imbalance data that cause two-class models to perform better. 

Additionally, this experiment was performed on a particular dataset of offensive utterances and conversational speech and generated positive outcomes in any derivation of the profanities. Nevertheless, the system performance may be altered by assessing a wider range of verbal words. Hence, more data samples involving the current nine profanities and other offensive words in films and entertainment shows should be gathered. Additionally, the proposed solution solely uses acoustic and frequency features of a given audio signal, and no language models were involved. Therefore, the proposed model could be used for different samples of another spoken term or another language provided the use of the same flow of data preparation to produce quality data that matches the proposed models and similar frequency feature extraction techniques. In this case, the models needed to adapt to the new and varying contexts of the training dataset for foul or normal classes that are different from the current dataset. Several strategies were recommended to tackle the study gap, such as providing full or partial system retraining with more samples or promoting transfer learning methods for CNN models [45]. Finally, a more detailed description of background speech sounds with specific labels (e.g., noises, music, and knocking) would be highly recommended to facilitate the immediate comprehension of speech samples and recognition under various backgrounds.

### 5.4. Impact of Noisy Data

In reality, offensive language identifications were aimed towards film censorship, such as spoken words that often occurred against noise-filled backgrounds or background music. Hence, the sturdiness of the system against noise was a crucial element. The integration of background noise to audios was performed using different SNRs. The model sturdiness was assessed using similar techniques with varying SNRs of 20 dB, 10 dB, and 0 dB, wherein the default samples were almost noise-free. The test was repeated with the same samples through five cross-validations and four varying sets of clean and noise-filled test samples. The accuracy of the assessment was based on k = 5 cross-validation for the four models. 

**Figure 3:**
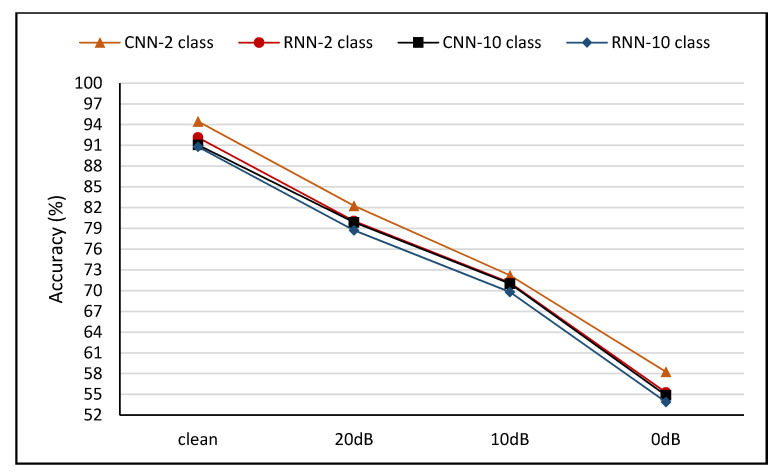
Impact of noisy data with non-augmented dataset.

For example, Figure 3 reveals that model accuracy decreased with the subsequent increase in background volume (lower SNR). Additionally, Figure 3 reflects the assessment of model accuracy using varying noise-related background volumes during the non-augmented dataset training. The best average performance was obtained by the CNN-class configuration with an accuracy of 94.47% through increased background noise. Meanwhile, accuracy fell to 58.28% at SNR = 0 dB with an average performance of 20 dB and 10 dB. The variance in decline between clean test samples and almost full noise samples (speech and background noise at the equal volume of 0 dB) denoted a difference in the accuracy of 39.19%. The accuracy drop variances for the remaining configurations were 36.19%, 36.86, and 36.90% for CNN-10 class, RNN-2 class, and RNN-10 class, respectively.

### 5.5. Impact of Data Augmentation

**Figure 4:**
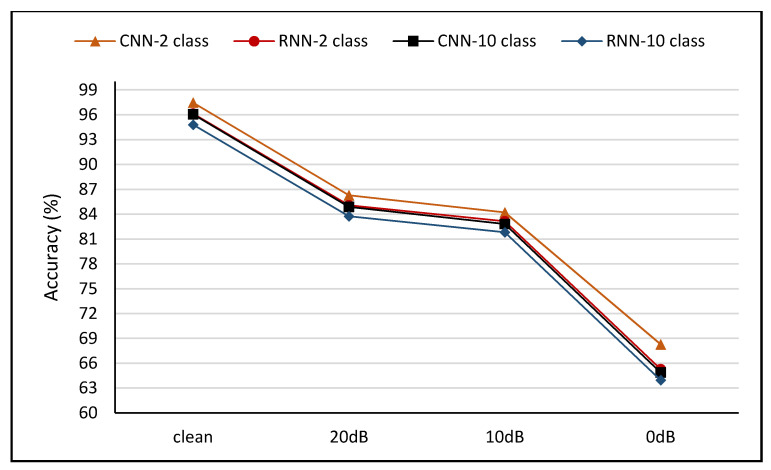
Impact of noisy data with augmented dataset.

In the study context, the offensive language identification tasks were extremely challenging following data scarcity in the particular field. Hence, data augmentation was applied in this study to increase the profane language dataset nine times and improve model sturdiness. The data augmentation impact was analyzed in the four cases testing model strength (clean, 20 dB, 10 dB, and 0 dB SNRs). The consequent improvement in performance and alleviation of accuracy differences through noise-filled environments are illustrated in Figure 4 and Table 10.

Figure 4 presents the assessment of all the model accuracies with various noise-filled background volumes during the augmented dataset training. Figure 4 also outlines the reduction in accuracy performance when the models were assessed using noise-filled test samples. Nonetheless, Figure 4 and Table 10 reveal that model performance was significantly elevated. For example, the CNN-2 class configuration accuracy improved by 3.01% with clean samples and by 10.10% with noise-filled samples at the SNR of 0 dB. Hence, data augmentation significantly elevated model performance.

Based on Figure 4 and Table 10, a noteworthy improvement was observed in model strength when assessed in noise-filled environments. Specifically, the difference in decline between clean and noise-filled test samples at the SNR of 0 dB was reduced to 29.21% (a variance of 9.98% between the decline in non-augmented and augmented data training) for CNN two-class configuration. The accuracy differences for other configurations were 31.09%, 30.17, and 30.86% for CNN-10 class, RNN-2 class, and RNN-10 class, respectively. The outcomes were lower than the difference in reduction between clean and noise-filled sample tests when trained with non-augmented data. Hence, training with augmented data elevated the strength of offensive language detection.

Table 10 presents the performance metrics for the four developed models based on accuracy trained with and without an augmented dataset while being tested with different levels of noise. Notably, data augmentation significantly improved the model performance and robustness. It was reported that the models trained with augmented data and tested with higher noise samples (10 dB SNR) produced better performance metrics than lower noise samples (20 dB SNR) when models were trained with a non-augmented dataset. For example, CNN-2 class at 10 dB SNR with data augmentation achieved 84.21% accuracy compared to the same model trained with non-augmented data at 20 dB SNR (82.28% accuracy).

## 6. Conclusions

This study suggested the implementation of two different ANNs (CNN and RNN models) for profane language identification in speech with pre-segmented test samples for automated film censorship. The collected dataset was manually labelled with two and 10 annotations. The CNN and RNN classifiers were trained to distinguish between the labels of pre-segmented incoming streams for offensive language identification. The suggested models performed positively in both the two-class and 10-class problems with FNRs ranging from 2.53% to 3.68% for two-class and 3.19% to 5.92% for 10-class. Proposed models achieved positive performance in terms of F1-score that comprises both precision and recall, where average F1-score achieved ranges from 97.56% to 96.73% for two-class and 96.11% to 94.85% for 10-class. Furthermore, CNN outperformed RNN for the same problem classes and affirmed a strong visual feature extraction in CNN. 

The proposed lightweight CNN model outperformed baseline algorithms of pre-trained networks in terms of performance metrics and model’s test speed attested by the small number of networks and test times using CPU and GPU. Proposed lightweight CNN outperformed baseline algorithms by about 1.91% F1-score and 1.57% FNR compared to the best performance of baseline algorithms, which is achieved by Resnet50. Model parameters required for our model are about 57 k, which is about 105 times less than GoogLeNet. This attests to the minimal test time taken for one sample prediction, which makes the proposed CNN model more favourable for real time application of monitoring, filtering, and censorship.

This study also suggested a unique dataset of offensive utterances and normal language. Following the impact of different noise levels and the data augmentation on model performance, a reduction in accuracy was observed involving noisier environments with the same test samples. Nevertheless, it was found that the decline in accuracy regarding model performance in the testing phase was alleviated. Augmented data samples in the training stage enabled the integration of noise with authentic and clean samples. Moreover, the study experiments were conducted on speaker-independent mode, whereby the test data samples of a specific speaker were not previously used in the training stage.

This study also demonstrated that CNNs (specifically used for visual identification) could be adapted to the categorization and identification of unfavourable spectral images from speeches. Thus, the viability of CNNs for both visual and speech identifications and censorship was affirmed. Most of the entertainment shows on television or video-sharing websites and applications involved visual, acoustic, and speech content and companies that required content censorship to provide functionality in identifying images or videos and speech or sounds. Thus, implementing the same technology for both censorship applications would remarkably reduce the developmental costs incurred.

## Figures and Tables

**Table 1:** Original data sample distributions of all classes.

Class	Train	Validation	Test
Foul/subclass	1863/207	621/69	621/69
Normal	3060	1020	1020

**Table 2:** Number of samples generated from different augmentation methods.

Class	Original Samples	Noise Incorporation	Frequency Manipulation	Pitch Manipulation	Total Samples
Foul/subclass	3105/345	12,420/1380	6210/690	6210/690	27,945/3105
Normal	5100	20,400	10,200	10,200	45,900

**Table 3:** Number of samples generated from different augmentation methods.

Layer Type	Number of Filters	Feature Map	Stride Value	Padding Value	Output
Image input layer		40 × 97 × 1			40 × 97 × 1
1st convolution layer ReLU Max pooling	12 12	3 × 3 × 1 3 × 3	[1 1] [2 2]	[1 1] [2 2]	40 × 97 × 12 40 × 97 × 12 20 × 49 × 12
2nd convolution layer ReLU Max pooling	24 24	3 × 3 ×12 3 × 3	[1 1] [2 2]	[1 1] [2 2]	20 × 49 × 24 20 × 49 × 24 10 × 25 × 24
3rd convolution layer ReLU Max Pooling	48 48	3 × 3 ×24 3 × 3	[1 1] [2 2]	[1 1] [2 2]	10 × 25 × 48 10 × 25 × 48 5 × 13 × 48
4th convolution layer ReLU	48	3 × 3 ×48	[1 1]	[1 1]	5 × 13 × 48 5 × 13 × 48
5th convolution layer ReLU Max pooling Dropout layer	48 48	3 × 3 ×48 1 × 13	[1 1] [1 1]	[1 1] 0	5 × 13 × 48 5 × 13 × 48 5 × 1 × 48 5 × 1 × 48
Fully-connected layer Softmax layer Classification layer	- -	- -	- -	- -	2 (10) 2 (10)

**Table 4:** Performance metrics of CNN two-class configuration.

Class	Precision (%)	F1-Score (%)	TPR (%)	FPR (%)
Foul	97.74 ± 1.19	96.92 ± 0.81	96.11 ± 2.65	2.19 ± 1.17
Normal	97.96 ± 1.09	98.39 ± 1.09	98.82 ± 1.02	2.17 ± 2.42
Average	97.85 ± 0.90	97.65 ± 0.90	97.47 ± 0.87	2.18 ± 0.70

**Table 5:** Performance metrics of RNN two-class configuration.

Class	Precision (%)	F1-Score (%)	TPR (%)	FPR (%)
Foul	96.49 ± 1.66	97.81 ± 1.36	99.17 ± 0.07	4.88 ± 1.66
Normal	98.32 ± 1.31	95.65 ± 1.08	93.12 ± 2.74	2.13 ± 3.31
Average	97.41 ± 0.92	96.73 ± 1.09	96.14 ± 1.08	3.51 ± 0.92

**Table 6:** Performance metrics of CNN 10-class configuration.

Class	Precision (%)	F1-Score (%)	TPR (%)	FPR (%)
A	99.59 ± 0.31	99.36 ± 0.46	99.16 ± 0.79	0.44 ± 0.69
B	96.79 ± 2.28	97.95 ± 1.34	99.16 ± 0.61	0.44 ± 0.67
Ba	97.57 ± 0.85	98.56 ± 0.70	99.58 ± 0.24	0.86 ± 1.32
Bi	91.17 ± 2.17	92.63 ± 1.85	94.16 ± 1.57	3.65 ± 4.84
C	90.27 ± 4.53	92.92 ± 3.19	95.83 ± 2.45	3.89 ± 4.01
Cu	96.87 ± 1.94	94.93 ± 2.78	93.33 ± 4.75	0.03 ± 1.94
D	94.55 ± 0.89	93.42 ± 2.12	92.60 ± 3.65	1.31 ± 2.58
F	97.21 ± 1.77	93.37 ± 3.16	90.00 ± 5.14	0.92 ± 1.74
P	99.59 ± 0.25	98.26 ± 1.12	97.08 ± 1.84	0.40 ± 0.75
N	99.68 ± 0.27	99.72 ± 0.17	99.77 ± 0.13	0.09 ± 0.61
Average	96.33 ± 1.06	96.11 ± 1.16	96.07 ± 1.22	1.20 ± 0.84

**Table 7:** Performance metrics of RNN 10-class configuration.

Class	Precision (%)	F1-Score (%)	TPR (%)	FPR (%)
A	98.28 ± 0.63	98.06 ± 0.62	97.86 ± 1.29	0.40 ± 0.61
B	95.49 ± 2.23	96.64 ± 1.51	97.86 ± 1.19	3.20 ± 0.63
Ba	96.27 ± 1.20	97.46 ± 0.60	98.69 ± 0.64	2.41 ± 0.70
Bi	90.26 ± 3.76	91.53 ± 3.43	92.86 ± 3.24	8.42 ± 4.01
C	88.97 ± 6.13	91.61 ± 5.96	94.52 ±6.45	4.44 ± 2.93
Cu	95.57 ± 4.01	93.62 ± 3.28	92.02 ± 4.21	3.12 ± 0.05
D	93.25 ± 3.06	92.12 ± 4.15	91.30 ± 6.71	5.44 ± 2.03
F	95.91 ± 2.82	92.06 ± 4.58	88.69 ± 6.65	2.78 ± 1.30
P	98.28 ± 0.63	96.96 ± 1.84	95.77 ± 3.70	0.40 ± 0.63
N	98.37 ± 0.32	98.42 ± 0.15	98.47 ± 0.03	0.31 ± 0.15
Average	95.06 ± 1.64	94.85 ± 1.71	94.80 ± 1.76	3.09 ± 1.91

**Table 8:** One-fold confusion matrix for RNN 10-class configuration.

	A	B	Ba	Bi	C	Cu	D	F	P	N
**A**	**576**	0	18	10	0	8	0	0	0	9
**B**	0	**620**	0	0	0	0	0	1	0	0
**Ba**	0	0	**621**	0	0	0	0	0	0	0
**Bi**	0	18	17	**495**	0	0	78	0	13	0
**C**	0	10	0	0	**558**	19	0	34	0	0
**Cu**	0	0	0	0	0	**621**	0	0	0	0
**D**	0	10	13	28	0	21	**459**	0	67	23
**F**	0	0	0	0	20	19	0	**558**	0	24
**P**	0	0	7	0	0	11	0	0	**603**	0
**N**	18	10	39	31	13	11	37	18	21	**8982**

**Table 9:** Comparative analysis of the developed model and the baseline models on MMUTM dataset.

Model/Reference/Year	FNR (%)	F1-Score (%)	Total Parameters	GPU Test Time (ms)	CPU Test Time (ms)
Fine-tuned Alexnet [11] (2020)	8.30	89.74	≈56 M	3.72	18.76
Fine-tuned VGG16 [11] (2020)	8.46	90.43	≈138 M	9.09	50.71
Fine-tuned GoogLeNet [11] (2020)	8.47	90.99	≈6 M	11.17	52.05
Fine-tuned Resnet50 [11] (2020)	5.49	94.20	≈23 M	19.86	97.38
**Proposed CNN Model**	**3.92**	**96.11**	**57 K**	**2.05**	**2.33**

**Table 10:** Model Performance based on accuracy (with and without data augmentation).

Model	Non-Augmented Data				Augmented Data			
	Clean	20 dB	10 dB	0 dB	Clean	20 dB	10 dB	0 dB
CNN-2 class	94.47	82.28	72.21	58.28	97.47	86.28	84.21	68.28
RNN-2 class	92.14	80.07	71.14	55.28	96.14	85.07	83.14	65.28
CNN-10 class	91.07	79.88	71.01	54.88	96.07	84.88	82.81	64.88
RNN-10 class	90.81	78.74	69.81	53.91	94.81	83.74	81.81	63.95

## Data Availability

The data presented in this study are currently available on request from the corresponding author. The data are not publicly available and will be available upon project MMUE/180029 completion.
